# A New Method for Determination of Pectin Content Using Spectrophotometry

**DOI:** 10.3390/polym13172847

**Published:** 2021-08-25

**Authors:** Furong Wang, Chao Du, Junjun Chen, Lisheng Shi, Hailong Li

**Affiliations:** School of Light Industry and Engineering, South China University of Technology, No.381 Wushan Road, Tianhe District, Guangzhou 510640, China; wangfurong199616@163.com (F.W.); 18816798517@163.com (C.D.); fecjj@mail.scut.edu.cn (J.C.); sls_shilisheng@163.com (L.S.)

**Keywords:** copper pectate, calcium pectate, ultraviolet and visible spectrophotometer

## Abstract

The study aimed at developing a new spectrophotometric method for determining the pectin content. Take commercial pectin as an example, and the method is based on the reaction of copper ions with pectin to produce copper pectate. The spectrophotometer was used to measure the remaining content of copper ions so as to calculate the pectin content. This method eliminated the weight step and avoided the error associated with it. Effects of reaction time, temperature, and pH on absorbance were also studied. Additionally, the accuracy of this method was verified. It indicated excellent repeatability and accuracy with the relative standard deviation of 2.09%. In addition, three different plant types were used to demonstrate the reliability of the method. To summarize, this method can be widely used for the determination of pectin content in many materials.

## 1. Introduction

Pectin is one kind of polymer with a complex chemical structure, which is mainly consisted of α-D-galacturonic acids [1]. As an important component of biomass, it is widely found in the intercellular layer and cell walls of plant cells [2]. Pectin is commonly used as a gelling agent [3], emulsifier, stabilizer [4], thickener, and heavy metal adsorbent [5] in the fields of food [6], medical, cosmetic, and other industries [7]. Rapid and accurate determination of pectin content is vital for improving its processing efficiency and optimizing the extraction method.

Currently, the pectin content determination methods typically include spectrometric method, chromatographic method, gravimetric method, etc. [8]. The mechanism of spectrometric method and chromatographic method is that hydrolysis products such as monosaccharides or galacturonic acid of pectin in acidic conditions could be measured by instruments [9,10]. Though they possess the advantages of high resolution and fast analysis speed, some complex pretreatment of samples is required [11], and other components such as cellulose and hemicellulose can also be hydrolyzed causing higher absorbance [12]. Most importantly, these methods must decompose pectin, therefore making it hard to retain it, so it is only slightly applied in some fields. Gravimetric method has been used widely the most. It is based on the chelation reaction of pectin with metal ions (Ca^2+^, Cu^2+^, Fe^3+^, Mg^2+^, etc.) to produce precipitation [13]. The content of pectin can be measured by calculating the weight of the precipitation. This process is complicated and time-consuming, although it can acquire the result directly. In the meantime, there are materials in which the amount of pectin is scarce and researchers must be extremely precise in order to reduce the error [14].

Based on the mechanism of gravimetric method, a novel method for determination of pectin content by spectrophotometry was proposed in this paper. By measuring the change of metal ion content before and after the reaction, the precipitation weight of pectin was calculated to obtain the content of pectin. Compared with the gravimetric method, it has the characteristics of higher efficiency and less error. In contrast with the spectrometric method and chromatographic method, the complex sample pretreatment process is eliminated so the pectin could be retained, and the reagents used in this method are less toxic and environmentally friendly. In a word, the method is effective, precise and accurate, which provides a new direction for the determination and application of pectin content in many materials.

## 2. Materials and Methods

### 2.1. Materials

Bark of paper mulberry; Wikstroemia; Sisal; Pectin: Aladdin, Shanghai, China; NH_3_·H_2_O: Guangzhou Chemical Reagent Factory, Guangzhou, China; NaOH: Guangzhou Chemical Reagent Factory, Guangzhou, China; CH_3_COOH: Tianjin Damao Chemical reagent Factory, Tianjin, China; CuCl_2_: Tianjin Damao Chemical reagent Factory, Tianjin, China; Na_2_CO_3_: Tianjin Damao Chemical reagent Factory, Tianjin, China; NaHCO_3_: Tianjin Damao Chemical reagent Factory, Tianjin, China. All the reagents used were obtained from commercial sources and the experimental water was distilled water.

UV-Vis spectrophotometer: UV-1900, Shimadzu, Japan.

FT-IR Spectrometer FTIR: Nicolet IS50—Nicolet Continuum, Thermo Fisher Scientific, Waltham, MA, USA.

Electro- Thermostatic Blast Oven: DHG-9070A, Shanghai Shenxian Constant temperature equipment Factory, Shanghai, China.

Electro-Thermostatic Water Bath: HH-6, Changzhou Aohua instrument, Changzhou, China.

### 2.2. Experimental Methods and Procedures

#### 2.2.1. Precipitated Pectin

The precipitation of calcium pectate and copper pectate was according to the precipitation of calcium pectate in GB/T 10742-2008 [15]. Among them, the precipitators were calcium chloride and copper chloride.

#### 2.2.2. Pectin Determination with Gravimetric Method

The filter paper with calcium pectinate (or copper pectinate) precipitation in Section 2.2.1 was placed in a flat weighing flask. Then, the flat weighing flask was moved into oven and dried to constant weight at 105 °C. In the meanwhile, the moisture content and the dry weight of filter paper were calculated. The content of pectin was expressed by the weight of calcium pectate (or copper pectate). The calculation formula is as follows:

(1) $$X=\frac{\left(m_{1}-m\right)}{m_{0}\left(100-w\right)}\times 100\%$$

where X is pectin content (measured by calcium pectinate or copper pectinate, in %); m is the absolute dry weight of the filter paper (in g); m_1_ is the total weight of dried calcium pectate (or copper pectinate) and filter paper (in g); m_0_ is the weight of the sample (in g); and W is the moisture of the sample (in %).

#### 2.2.3. Pectin Determination with Copper Ion Colorimetry Method

1. Establishment of Cu^2+^ Working Curve

Two milliliters of different concentrations (3~10.5 mmol/L) of CuCl_2_ solution were put into a set of 10 mL of the volumetric flask containing buffer solution, respectively. The resulting solution was transferred to the quartz cuvette, and buffer solution was used as a blank sample. The absorbance at 712 nm was determined by a UV-Vis spectrophotometer. A linear standard curve was established between the absorbance and the content of Cu^2+^.

2. Determination of Pectin Content

Two milliliters of filtrate in Section 2.2.1 steps was added into 10 mL of the volumetric flask, which contains 8 mL of buffer solution. After mixing well, the mixture was moved to a quartz cuvette, and the absorbance at 712 nm was measured in a UV-vis spectrophotometer to determine the content of Cu^2+^ in the filtrate, which can further be used to calculate the pectin content. The calculation formula is as follows:

(2) $$X=\frac{\left(n_{0}-c\times V\times 10^{-3}\right)\times 64}{0.1172\times m_{0}\times \left(100-w\right)\times 10^{3}}\times 100\%$$

where X is pectin content (measured by copper pectate content, in %); C is the concentration of Cu^2+^ according to the working curve (in mmol/L); n_0_ is the content of Cu^2+^ before the reaction (in mmol); V is the total volume of the filtrate (in mL); 64 is the relative atomic weight of copper; 0.1172 is the ratio of copper atoms in copper pectate; m_0_ is the weight of the sample (in g); and W is the moisture of the sample (in %).

## 3. Results

### 3.1. Sample Analyses

**Figure 1:**
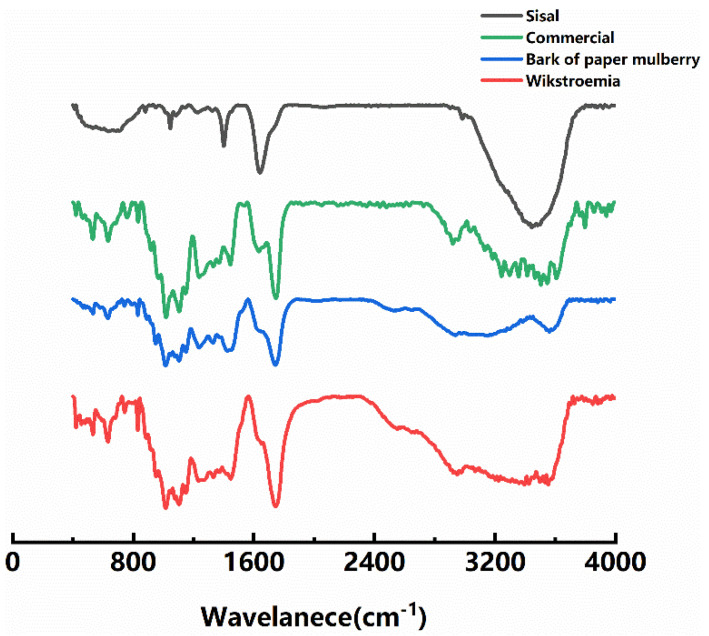
FT-IR spectra of different pectin types.

The chemical characteristics of several different pectin types were analyzed by FT-IR, and the results are shown in Figure 1. The wide peak between 2300 and 3800 cm^−1^ is the result of O–H stretching vibration of intramolecular or intermolecular hydrogen bonds, and the vibration of pectin is mainly caused by intramolecular and intermolecular hydrogen bonds of galacturonic acid polymers. The weak absorption peak near 2930~2980 cm^−1^ is caused by the stretching vibration of C–H (including CH, CH_2_ and CH_3_) of sugars. For pectin samples, the residue of methyl galacturonic acid produces O–CH_3_ stretching vibration between 2950 and 2750 cm^−1^. The absorption peak near 1745 cm^–1^ is the stretching vibration of the ester bond (–COOR) C–O formed by the carboxyl aldehyde group. The absorption peak at 1630 cm^−1^ is the asymmetric stretching vibration of free carboxylic acid or carboxylic acid salt (–COO) C=O, and is also the absorption peak of the sugar hydrate sample. The absorption peak of 1300~1000 cm^−1^ is caused by the stretching vibration of C–O, one of which is attributed to C–O–H on the sugar ring and C–O–C glycoside bond, and the other is attributed to C–O–H and C–O–R of the carboxyl galacturonic acid group [16].

Meanwhile, the degree of esterification (DE) was determined using FTIR analysis method. Results are shown in Table 1. It can be seen that the pectin samples we used included both LMP(DE < 50%, Low Methoxyl pectin) and HMP(DE ≥ 50%, High Methoxyl pectin). DE is a ratio of methyl-esterified carboxyl groups to the number of total carboxyl groups present. The percentage of DE was determined according to Equation (3) [17]:

(3) $$\mathrm{DE}(\%)=\frac{A_{1745}}{A_{1745}+A_{1630}}\times 100$$

where A_1630_ and A_1745_ denote the absorbance intensity, respectively, at 1630 and 1745 cm^−1^ for non-methyl-esterified carboxyl groups and the methyl-esterified carboxyl groups.

### 3.2. The Mechanism of the Copper Ion Colorimetry Method

**Figure 2:**
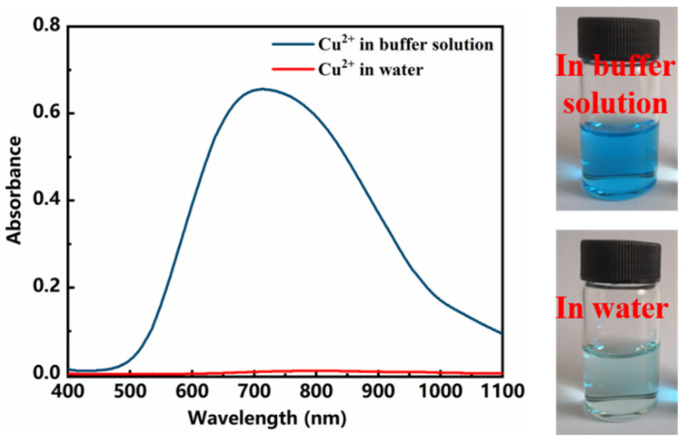
Absorption spectra for Cu^2+^ in water and in buffer solution.

When determining pectin content with the gravimetric method, Ca^2+^ is used as the precipitant during the process of a precipitation reaction. However, the absorbance of Ca^2+^ is difficult to determine unless the addition of a certain number of chromogenic agents [18,19,20]. Even in this situation, its absorbance should be measured after a period of time in a dark environment. This process is complicated, and the absorbance fluctuates greatly. Therefore, Cu^2+^ is selected as the metal ion to be measured. In addition, Cu^2+^ could react with buffer solution to produce basic copper carbonate, as shown in below reaction, which has the specific absorption peak around 710 nm [21]. Figure 2 showed the spectrum of Cu^2+^ in buffer solution and water, respectively.

2Cu^2+^ + CO_3_^2-^ + 2OH^−^ = Cu_2_(OH)_2_CO_3_

**Figure 3:**
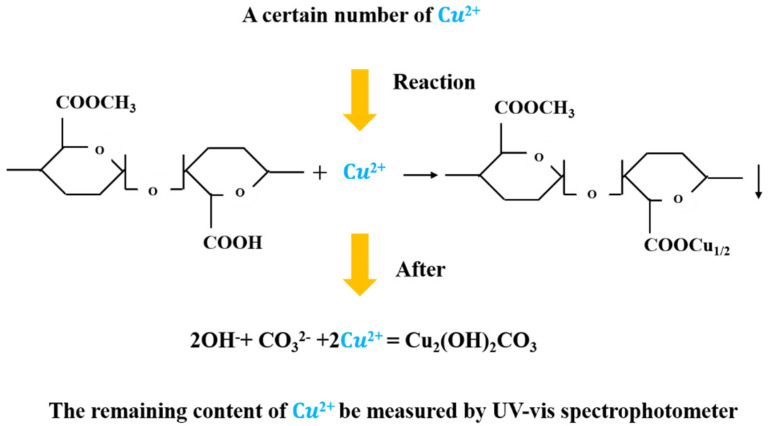
Mechanism of pectin content determination by copper ion colorimetry.

The molecular formula of copper pectate is C_17_H_22_O_16_Cu, where Cu accounts for 11.72% of the weight of copper pectate [22,23]. Therefore, the content of copper pectate can be obtained by measuring the content of Cu^2+^, which can be calculated by measuring the Cu^2+^ content in the filtrate. Based on this mechanism (as shown in Figure 3), a new method for the rapid determination of pectin content was established in this paper.

### 3.3. Effects of Process Parameters on Absorbance of Basic Copper Carbonate

#### 3.3.1. Determination of Optimal Wavelength

**Figure 4:**
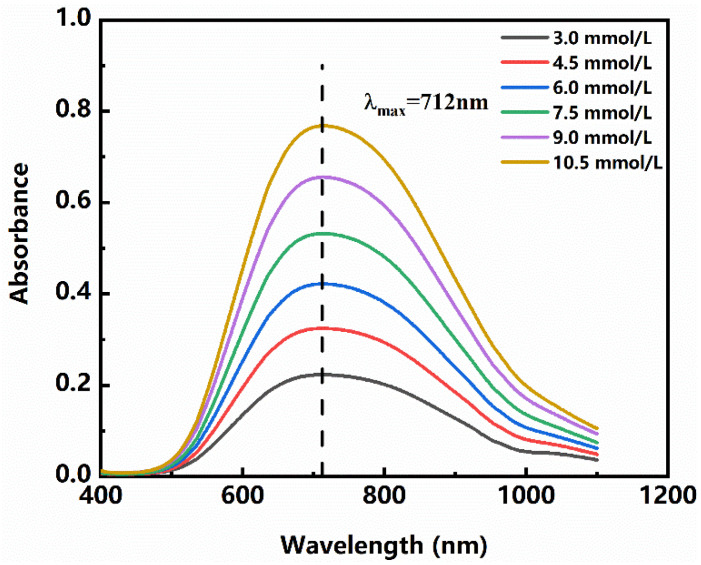
Absorption spectra of Cu^2+^ with different concentrations in buffer solution.

Absorption for different concentrations of Cu^2+^ is presented in Figure 4. It serves to show that the solution has an absorption maximum at 712 nm, which is consistent with the literature [24]. As the concentration of Cu^2+^ changes, the absorbance of its absorption maximum peak has a good linear relationship and conforms to the Beer–Lambert Law [25]. Therefore, 712 nm is selected as the determination wavelength in this paper.

#### 3.3.2. Effect of Reaction Time

**Figure 5:**
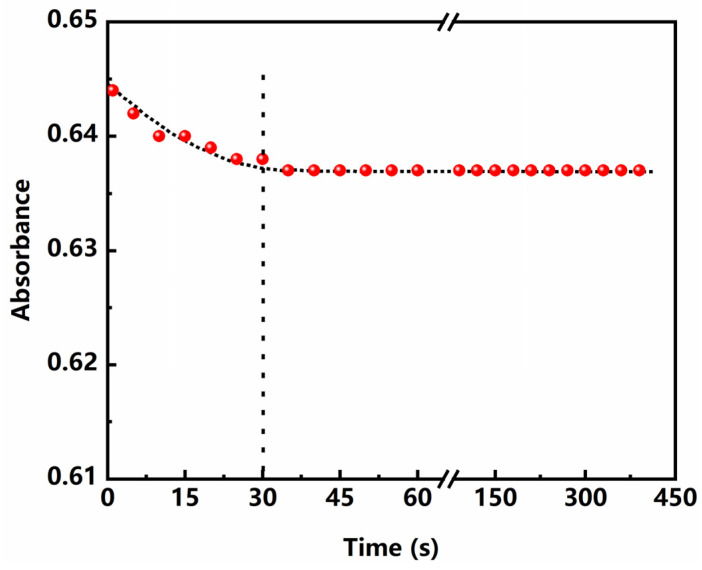
Effect of reaction time on the absorbance.

The effect of reaction time on the absorbance is shown in Figure 5. It can be seen from the figure that the absorbance of the reaction solution decreased gradually at first and then achieved equilibrium. It was observed that a minimum of 30 s was required for complete conversion of Cu^2+^ to basic copper carbonate. Therefore, the buffer solution was added and stayed for 30 s to determine in this experiment.

#### 3.3.3. Effect of Reaction Temperature

**Figure 6:**
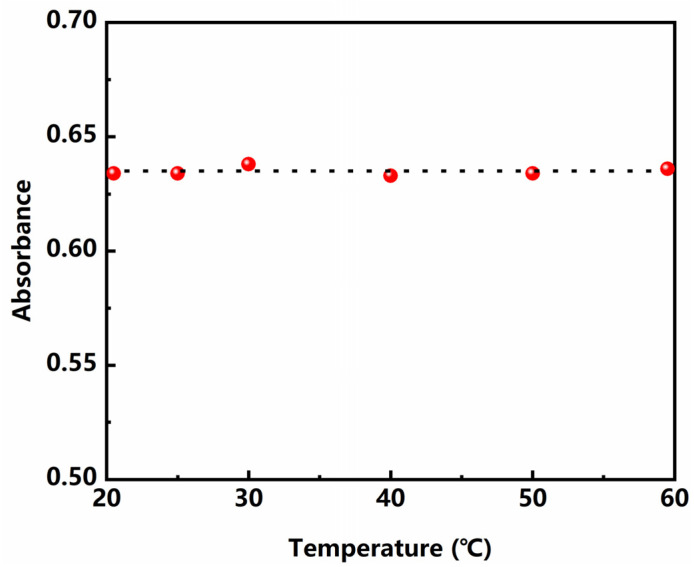
Effect of temperature on the absorbance.

Figure 6 represents the absorbance changing with reaction temperature at 712 nm for basic copper carbonate. It can be seen that the absorbance remains stable when the reaction temperature increased from 20 to 60 °C, indicating that the reaction system has good stability. All the experiments in this study were conducted at room temperature.

#### 3.3.4. Effect of pH

**Figure 7:**
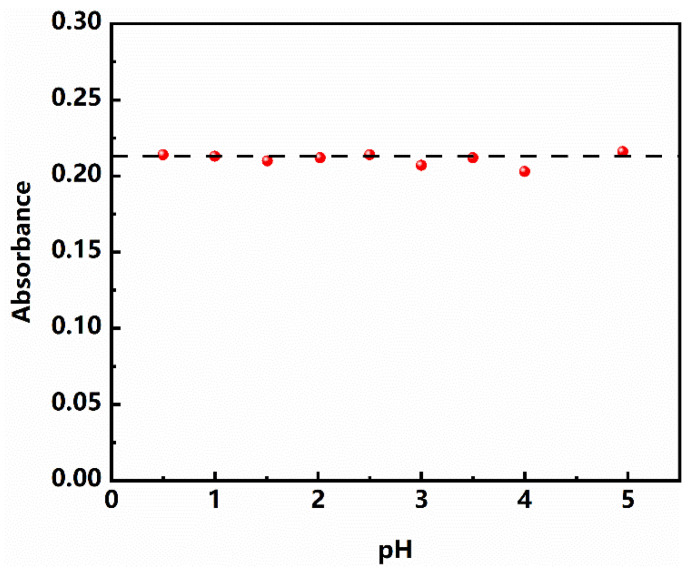
Effect of pH on the absorbance.

Before the determination of pectin content, the technique of “acid extraction and alcohol precipitation” is usually used to extract pectin [26,27]. Different extraction methods will lead to different degrees of acidification of the solution [28]. Therefore, the relationship between pH and absorbance in solution was investigated. The initial pH of the solution was 4.95, and hydrochloric acid was added to adjust the solution to different pH values, as shown in Figure 7. It can be seen that the absorbance of the reaction system does not change significantly under different pH conditions, which indicates that the change of pH does not affect the determination of pectin content.

### 3.4. Establishment of Working Curve

**Figure 8:**
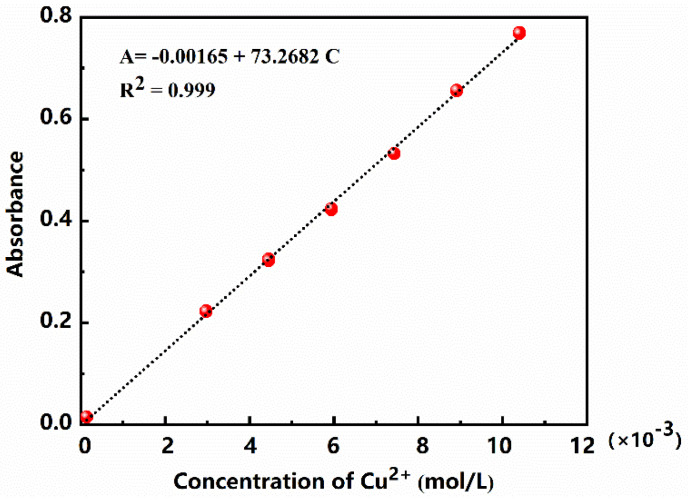
Working curve of Cu^2+^.

The working curve of the relationship between concentration of Cu^2+^ and the absorbance at 712 nm was measured and established in accordance with 2.2.3 steps. Results are shown in Figure 8. The equation of the working curve can be expressed as:

*A* = −0.00165 (±0.00534) + 73.2682 (±0.778) × *C*, (R^2^ = 0.999) (4)

i.e.,

A = *a*(Δ*a*) + *s*(Δ*s*) × *C* (5)

where *C* is the concentration of Cu^2+^ in the system (in mol/L), while *A* represents the absorbance of the solution at 712 nm. The limit of quantitation (LOQ) is generally evaluated by the following Equation (6) [29]. The calculated value of LOQ is 0.706 mmol by using the method that the paper referred, which is equivalent to the pectin content of 0.385 g/L.

LOQ = (*a* + 10|Δ*a*|)/*s* (6)

### 3.5. Interference

In order to explore the influence of other components in the filtrate system, four portions of filtrate in Section 2.2.1 steps were taken and different concentrations of CuCl_2_ standard solution were added, respectively. The Cu^2+^ concentration was determined according to Section 3.4 steps, and the recovery rate [9] was calculated. As is shown in Table 2, the recovery rate of the experimental samples ranged from 96.04% to 98.39%, suggesting that other components in the filtrate system have little influence on the results. That is to say, the method has good accuracy and meets the requirements of determination.

### 3.6. Method Calibration and Evaluation

In order to verify the accuracy and precision of this method, the repeatability of the method was analyzed, its relative standard deviation (RSD) in five measurements is 2.09%, indicating that the method has good repeatability and high precision.

Four kinds of different pectin content samples were used to verify the feasibility and reliability of this method and the results are shown in Table 3. It can be seen that the pectin content measured by this method was closed to the gravimetric method. The RSD are less than 3%, indicating that this method is reliable for pectin content determination.

### 3.7. Universality of the Method

In order to validate the universality of the method, copper ion colorimetry method and gravimetric method were used, respectively, to determine pectin content of several different plant sources. The bark of paper mulberry, sisal, and wikstroemia were chosen as raw materials. Results are shown in Table 4, where it can be seen that for different plant materials, copper ion colorimetry method had insignificant differences compared with the gravimetric method. The RSD between them is within 5.00% and decreased with the increase of pectin content. In conclusion, the copper ion colorimetry method has the characteristics of universality, and it can be widely used in the determination of pectin content of plant raw materials.

## 4. Conclusions

This paper presents a novel method for the determination of pectin content. The content of pectin was determined by UV-vis spectrophotometer after stabilization of 30 s at room temperature. Results showed that the method has a considerable measurement precision and accuracy (RSD = 2.09%). Compared with gravimetric method, the RSD of pectin content measured by them is less than 3%. In conclusion, the method is simple, accurate and repeatable. It can be widely used in the determination of pectin content, which provides a new idea for the determination of pectin content and the application of pectin.

## Figures and Tables

**Table 1:** DE of different pectin types.

Samples	DE (%)
Commercial	40.35
Sisal	57.37
Bark of paper mulberry	44.06
Wikstroemia	35.81

**Table 2:** Recovery rate of Cu^2+^.

Samples	Original Measurements (µmol)	Addition(µmol)	Measurements After Addition(µmol)	Recovery Rate(%)
1	44.2	14.9	58.7	97.22
2	41.9	22.3	63.3	96.04
3	42.0	44.7	86.0	98.39
4	37.3	29.8	66.2	97.22

**Table 3:** Comparison of copper ion colorimetry method and gravimetric method.

Samples	Gravimetric Method (g)	Copper Ion Colorimetry Method (g)	RSD (%)
1	0.6233	0.6463	2.60
2	0.9613	0.9763	1.10
3	4.6193	4.6102	0.14
4	4.9617	4.9399	0.31

**Table 4:** Comparison of copper ion colorimetry method and gravimetric method for different pectin types.

Samples	Gravimetric Method (g)	Copper Ion Colorimetry Method (g)	RSD (%)
Bark of paper mulberry-1	1.1204	1.0679	3.40
Bark of paper mulberry-2	1.3354	1.4033	3.51
Sisal-1	0.4920	0.4590	4.90
Sisal-2	0.5528	0.5278	3.28
Wikstroemia-1	2.4874	2.5103	0.65
Wikstroemia-2	3.7498	3.7156	0.65

## Data Availability

Not applicable.
